# Successful Anesthetic Management of Video-Assisted Thoracic Surgery in a Patient With Cardiac Complications After Arterial Switch Operation

**DOI:** 10.7759/cureus.88547

**Published:** 2025-07-22

**Authors:** Yasuhiro Watanabe, Yuki Kato, Chizuru Ishida, Takamitsu Hayakawa, Kayoko Matsunuma

**Affiliations:** 1 Department of Anesthesia, Japanese Red Cross Shizuoka Hospital, Shizuoka, JPN; 2 Department of Thoracic Surgery, Japanese Red Cross Shizuoka Hospital, Shizuoka, JPN

**Keywords:** adults with congenital heart disease, arterial switch operation, hypoxic pulmonary vasoconstriction, one-lung ventilation, pulmonary stenosis, transposition of the great arteries, video-assisted thoracic surgery

## Abstract

The arterial switch operation with the Lecompte maneuver has become the therapy of choice for dextro-transposition of the great arteries, contributing to the improved survival rate of patients. Nevertheless, there have been few case reports addressing anesthetic management using one-lung ventilation in patients with an arterial switch. A 24-year-old man who underwent an arterial switch operation at 10 days of age was admitted for a right spontaneous pneumothorax. Preoperative transthoracic echocardiography revealed significant neo-aortic regurgitation, tricuspid regurgitation, and estimated right ventricular systolic pressure at the upper normal limit, all of which can be observed after the procedure. The pulmonic valve and pulmonary artery were difficult to delineate by transthoracic echocardiography. Instead, a plain CT demonstrated non-stenotic pulmonary arteries. During left isolated ventilation in the lateral decubitus position, the patient's hemodynamics and oxygenation were both well maintained, and a right thoracoscopic bullectomy was performed uneventfully under total intravenous anesthesia with propofol and remifentanil. Vital signs were also stable postoperatively, and the patient was discharged on the fifth postoperative day without adverse cardiovascular events. In the present case, the non-stenotic left pulmonary artery was considered to have played a vital role in successful one-lung ventilation, highlighting the importance of multimodal evaluation for long-term cardiac complications in a patient who underwent an arterial switch operation.

## Introduction

Remarkable progress in surgical technique and medical care has dramatically improved the survival rate of infants with congenital heart disease (CHD) [1]. The number of adults with CHD (ACHD) continues to increase in Japan [2], as well as in other developed countries [3,4]. It was reported that the proportion of ACHD undergoing non-cardiac surgery increased by 2.6 times between 2002 and 2009 [5]. Ample evidence indicates that anesthesiologists might be increasingly involved in the perioperative management of ACHD.

Dextro-transposition of the great arteries (d-TGA) is a once-fatal cyanotic heart disease that accounts for approximately 5% of all CHD cases and 10% of neonatal cyanotic CHD cases [6]. The arterial switch operation corrects ventriculo-arterial discordance, in which the aorta and main pulmonary artery (PA) are transected above each sinus and translocated to the opposite root, with reimplantation of the coronary arteries into the neo-aorta. The PA is placed in front of the aorta using the Lecompte maneuver to prevent distortion [7]. Over the past three decades, the arterial switch operation has replaced atrial switch procedures as the surgical therapy of choice for d-TGA. Accordingly, the number of patients with an arterial switch is steadily growing [8]. Meanwhile, few clinical reports have addressed cases of one-lung ventilation (OLV) in patients with an arterial switch. Herein, we report successful anesthetic management of video-assisted thoracic surgery in a patient complicated with long-term cardiac lesions after an arterial switch operation.

## Case presentation

**Figure 1:**
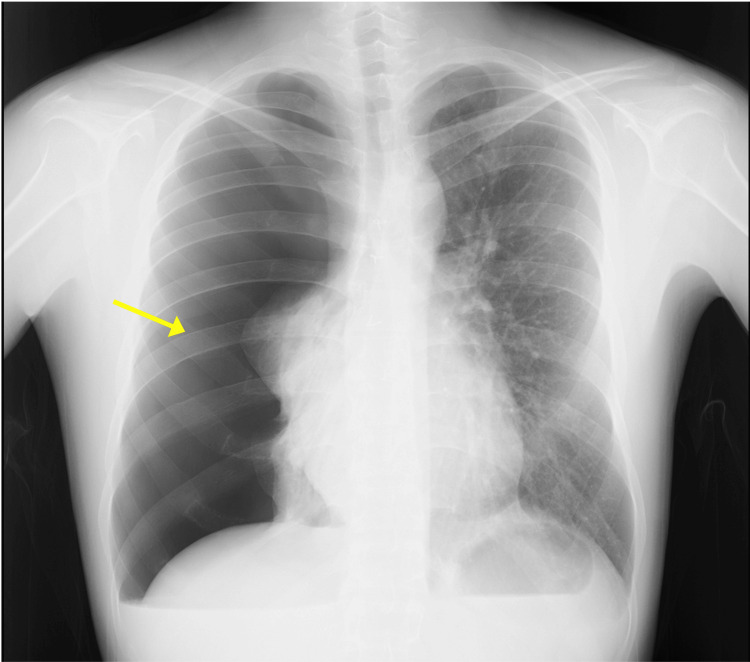
Chest radiograph acquired at the emergency department A massive right pneumothorax (yellow arrow) with a cardiothoracic rate of 41.9% is observed in the upright position.

A 24-year-old man (height 172 cm; weight 66 kg; body mass index 22.3 kg/m²) was admitted due to acute-onset right chest pain and dyspnea. Vital signs at the emergency department demonstrated blood pressure of 140/83 mmHg, heart rate of 75 beats per minute (bpm), body temperature of 37.0°C, respiratory rate of 20 per minute, and percutaneous oxygen saturation (SpO_2_) of 90% in room air. Chest radiography revealed a massive right-sided pneumothorax (Figure 1), requiring the insertion of a thoracic drainage catheter. 

**Figure 2:**
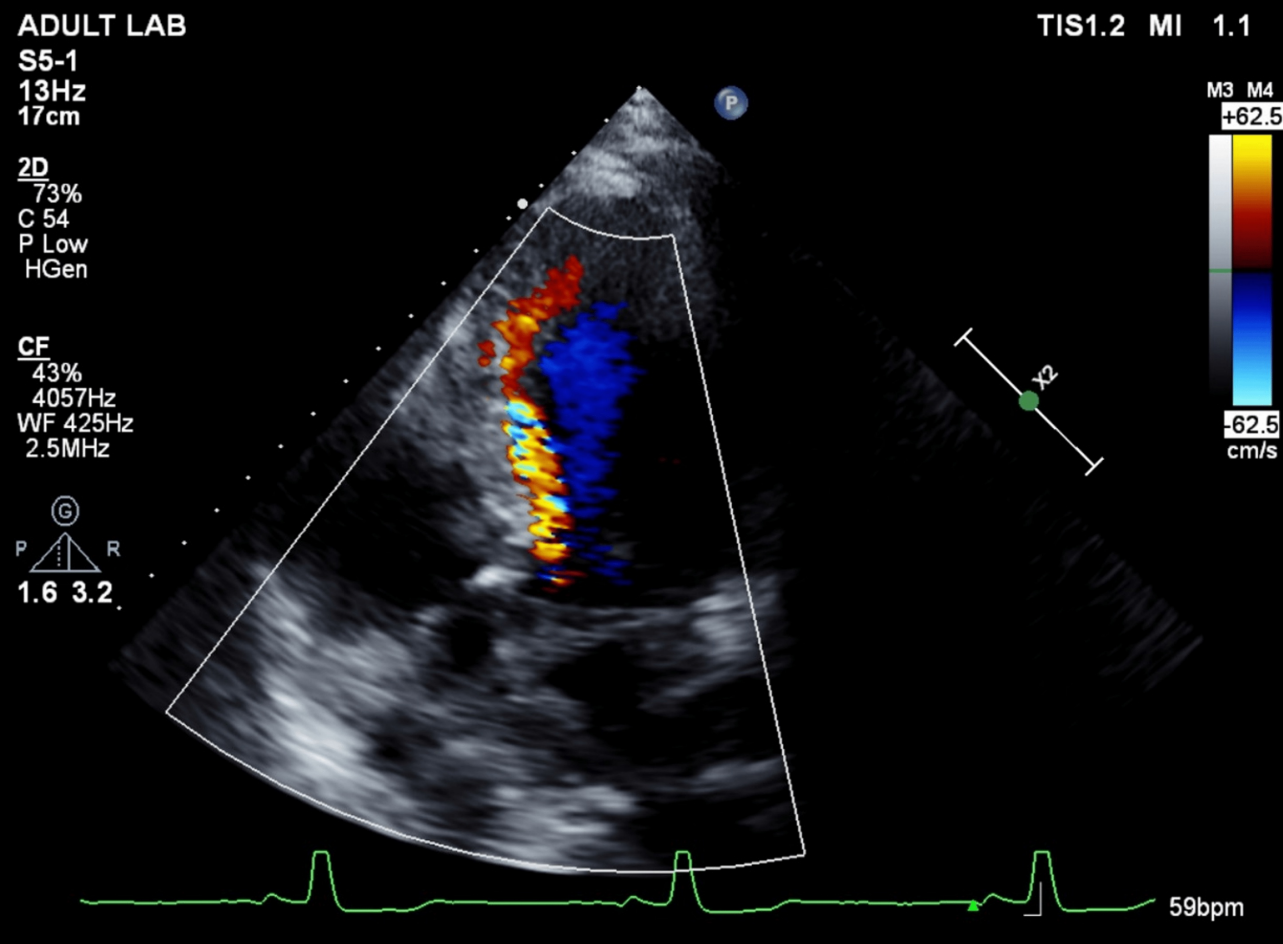
Color Doppler imaging demonstrating neo-aortic regurgitation The color Doppler imaging in an apical four-chamber view depicts a moderate-to-severe degree of neo-aortic regurgitation, which is narrow and deviated, nearly reaching the apex.

**Figure 3:**
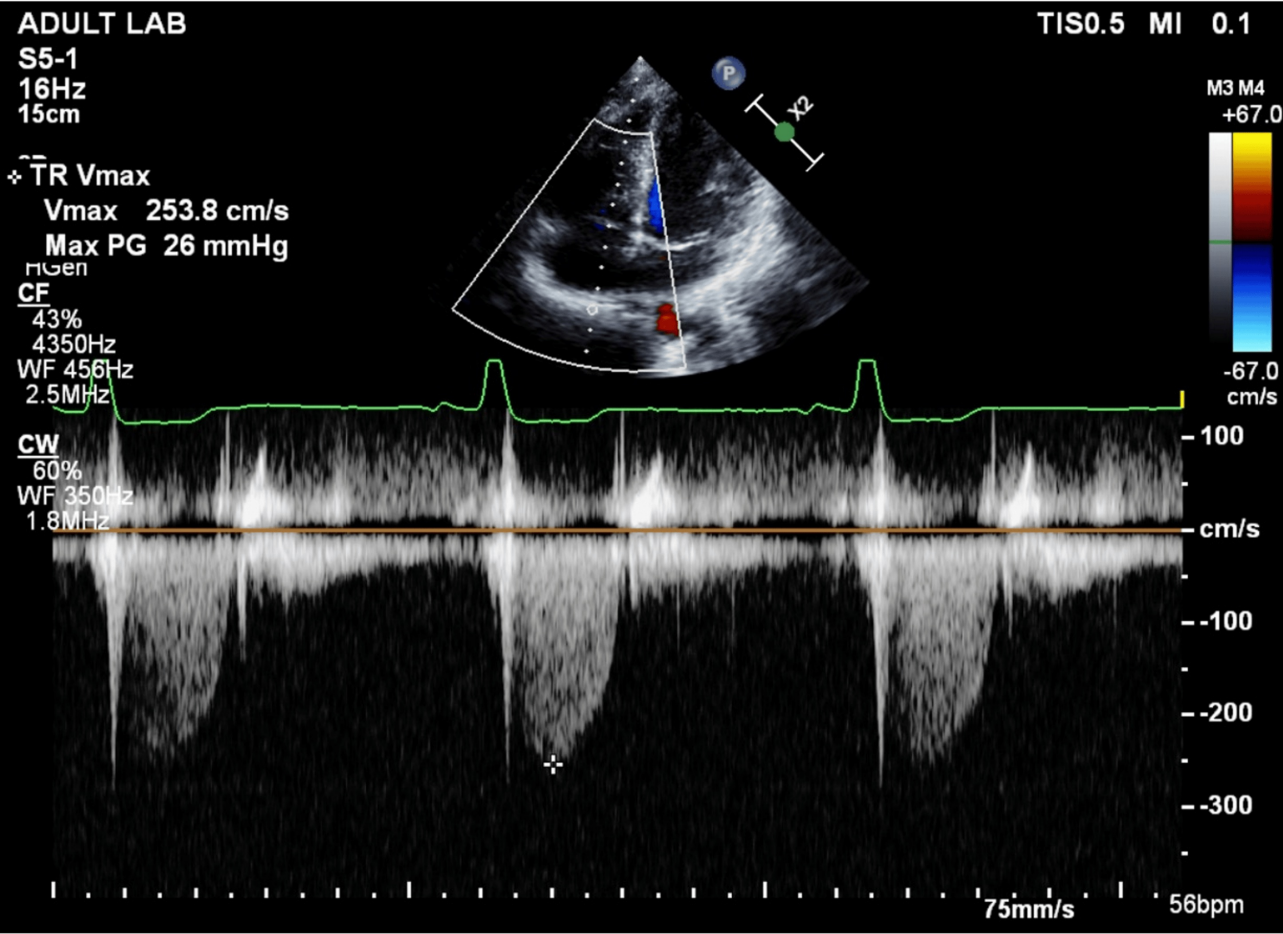
Continuous-wave Doppler echocardiography of tricuspid regurgitation suggesting a more than mild degree of regurgitation

**Figure 4:**
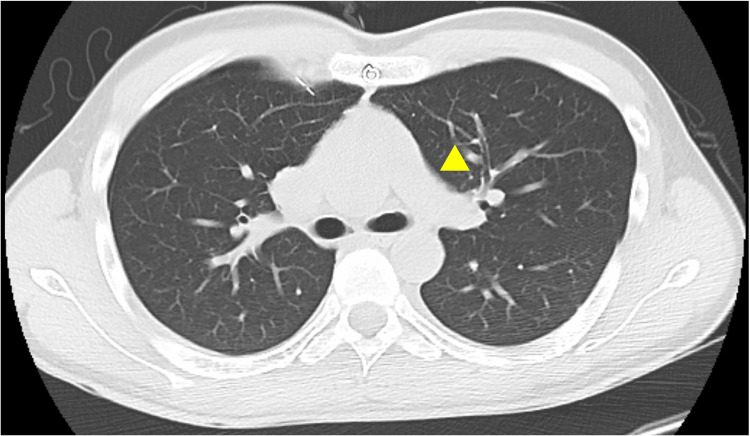
CT of the great arteries The PA lies in front of the aorta, suggesting that the patient had undergone an arterial switch operation using the Lecompte maneuver. Although not contrast-enhanced, no obvious stenosis of the left PA is observed (yellow arrowhead). PA: Pulmonary artery

A CT performed three days after admission showed expansion of the lung; however, no further improvement was expected, and a thoracoscopic bullectomy was scheduled three more days later. The patient underwent an arterial switch operation using the Lecompte maneuver for d-TGA at 10 days of age and attended a specialized outpatient clinic once a year with no medication. There was no restriction in his activities of daily living, and the Revised Cardiac Risk Index scored zero. Electrocardiography revealed normal sinus rhythm with no ST-T changes, and the transthoracic echocardiography (TTE) performed two days prior to surgery showed neither wall motion abnormalities nor diastolic dysfunction of the left ventricle, with an ejection fraction of 62%. These findings were suggestive of intact coronary circulation. In contrast, moderate-to-severe neo-aortic regurgitation (Figure 2), mild mitral regurgitation, and mild-to-moderate tricuspid regurgitation (Figure 3) were observed. The diameter of the inferior vena cava was 14 mm, accompanied by >50% collapse with sniff, suggesting the right atrial pressure was 0-5 mmHg, and the estimated right ventricular systolic pressure (RVSP) was 29 mmHg, within the upper normal limit of 30-35 mmHg. Dilatation of the neo-aortic root was also detected. The pulmonic valve and PA were difficult to delineate by TTE. Instead, no obvious stenosis of the PA was observed in plain chest CT (Figure 4). Blood test results were all normal except for the international normalized ratio of prothrombin time of 1.16.

In addition to the standard equipment (non-invasive intermittent blood pressure measurement, electrocardiography, SpO_2_, end-tidal carbon dioxide (EtCO_2_), and body temperature), anesthetic depth (BIS™; Medtronic, Minneapolis, MN, USA) was monitored. Vital signs in the operating room showed blood pressure of 128/77 mmHg, heart rate of 77 bpm, and SpO_2_ of 95% in room air with an inserted thoracic drainage catheter. Due to difficulty in securing a peripheral venous line, general anesthesia was induced with sevoflurane inhalation and subsequently switched to a target-controlled infusion of propofol (2.5 μg/mL) and remifentanil (0.4 μg/kg/min). Immediately after tracheal intubation with a 32-Fr left-sided double-lumen tube, left OLV was initiated with pressure-controlled ventilation with a peak pressure of 20 cmH₂O, positive end expiratory pressure (PEEP) of 5 cmH₂O, and a fraction of inspired oxygen (FiO₂) of 1.0. Eight minutes later, FiO₂ was decreased to 0.7 and continued until the end of OLV, during which SpO₂ was maintained above 99%. Anesthesia was maintained with propofol (2.0-2.5 μg/mL), remifentanil (0.1-0.2 μg/kg/min), and intermittent administration of fentanyl and rocuronium by referring to the anesthetic depth. Only three bolus administrations of ephedrine were required to maintain a systolic blood pressure of 90 mmHg throughout the surgery. Intraoperatively, electrocardiography showed normal sinus rhythm with a heart rate of 60-80 bpm, and EtCO₂ was maintained between 38 mmHg and 40 mmHg during OLV. Right thoracoscopic bullectomy was performed uneventfully in the left decubitus position, and the patient was extubated after the reversal of muscle relaxation with sugammadex.

The duration of operation and OLV was 73 minutes and 83 minutes, respectively, and anesthesia lasted 139 minutes. The patient lost 5 mL of blood, produced 200 mL of urine, and received 800 mL of crystalloid infusion. Vital signs were also stable postoperatively, and the patient was discharged on the fifth postoperative day without adverse cardiovascular events. 

## Discussion

Adults with CHD who undergo non-cardiac surgery have higher perioperative morbidity and mortality rates compared with their matched cohorts [5,9,10]. In addition to age-related disorders, as ACHD grow older, the chronic consequences specific to surgical repair or primary lesions may worsen, requiring a more careful anesthetic approach. Practically, it is recommended that either cardiac MRI or cardiac CT should be acquired every 12 to 36 months in patients who underwent arterial switch operations [11]. In the present case, TTE revealed several cardiac complications that are typically observed after arterial switch operation. Of note, the structures of the right heart system were difficult to delineate by TTE; instead, plain CT which was performed to assess the expansion of the lung, was also useful in the evaluation of the central PA. 

In patients who've undergone arterial switch operation, despite excellent survival rates [12,13], long-term cardiac sequelae, including pulmonary stenosis, neo-aortic root dilatation, neo-aortic regurgitation, and coronary obstruction, are common [8,11]. Particularly, pulmonary stenosis remains the most frequent indication for reintervention [13-15], and over 85% of the lesions are supravalvular (i.e., PA) stenosis [14]. Moreover, invasive measurements showed that RVSP was 47 ± 15 and 65 ± 18 (mmHg, mean ± standard deviation) in patients with an arterial switch undergoing catheter intervention for unilateral and bilateral PA, respectively [16]. In our case, the echocardiographic RVSP, which reportedly shows a good correlation with invasive measurements [16], was 29 mmHg. Along with CT imaging, it was suggested that the PAs were not stenotic enough to require intervention. 

In thoracic surgery performed in the lateral decubitus position, pulmonary blood flow through the dependent lung increases up to 160% during OLV [17]. If the present patient had significant left PA stenosis, an increase in left pulmonary blood flow may have led to RV pressure overload, exacerbation of tricuspid regurgitation, and eventual circulatory compromise. Thus, we believe that the non-stenotic left PA contributed to the maintenance of hemodynamics and oxygenation during OLV. In the present case, propofol was used as a general anesthetic primarily due to its property of not inhibiting hypoxic pulmonary vasoconstriction in the non-dependent lung [18]. In any case, atelectasis of the dependent lung, which induces pulmonary vasoconstriction and leads to further elevation in RV afterload, should be avoided by applying an appropriate PEEP and lung recruitment maneuver.

## Conclusions

A right thoracoscopic bullectomy was successfully performed under total intravenous anesthesia in a patient with cardiac complications who had undergone an arterial switch operation. The non-stenotic left PA was thought to have played a vital role in successful OLV. The TTE is capable of demonstrating significant neo-aortic regurgitation, tricuspid regurgitation, and estimated RVSP at the upper normal limit. In contrast, chest CT is exclusively useful in clarifying non-stenotic central PA. Thus, acquisition of preoperative CT, even if not contrast-enhanced, can be crucial in evaluating the great arteries of a patient who underwent an arterial switch operation.
